# Everybody's Page

**Published:** 1888-11-24

**Authors:** 


### Everybody's Page.

It is said that Mr. Gladstone attributes his good health and long life to the fact that he makes twenty-five bites to each piece of meat he consumes. The proportion of bites to bread, the proverbial cherry and other things is not known.

Blindness is on the increase in America. While the population of the States increased 30 per cent. between 1870 and 1880, blindness developed at the enormous rate of 140 per cent. This rate seems incredible, but the statistics collected by Dr. Howe, of Buffalo, seem to prove its accuracy.

One of the most curious effects on the organism of the inhalation of sulphuric ether is what has been termed the "anæsthetic revelation." Just before full consciousness returns the patient has a vivid perception of what appears to him to be the secret of the universe, the true meaning of our existence ; its cause and end. It is strange that while the thoughts of the nearly drowned revert to the past, those of half unconscious experimenters with anæsthetics should peer into that which may be revealed in the future of the after world.

Mr. Hewitt, the Mayor of New York, has been doing good work for the insane. When he came into office he found that the cooking in the asylums was of a disgraceful nature. The chief cook, appointed for political and not gastronomic considerations, neglected his work, and the lunatics suffered. Mr. Hewitt took up the matter, got a proper cook-house built, and a competent chef appointed, under whom he placed a number of the patients, who have now learned to cook, and are the happier for having something to do. And while the lunatics profit in every way, the cost of working is less than under the old bad system.

Professor Waldeyer, of Berlin, read a paper on "Women, and the Study of Medicine;" to the recent meeting of the Society of German Naturalists and Physicians, in which he spoke in unflattering terms of the medical women. He said that science made but slight progress in the hands of women, even in those branches which might be supposed to be in a special degree woman's work, and he was so unkind as to say that in the laboratory, one female student caused as much trouble to the assistant as thirty men. If this be true, it is sad, but even if we accept the professor's statements without reservation, it is well to remember that though we cannot all be Waldeyers, there is work to be done, most useful and necessary work, by the rank and file of the profession, who are not more skilled than the average lady doctor. And perhaps if Germany held India among her possessions, she might see a use, nay, a necessity for medical women.

In connexion with the series of murders that is now occupying the attention of the London public, it is worth while recalling a similar set of crimes that took place in Texas in the year 1885. The victims were eight women, two of whom were white and the rest coloured. The murderer has never been discovered, but that one person was guilty of all seems likely from the fact that there was a great similarity among the crimes. All the victims were killed about midnight, usually within a few days of full moon ; they were struck with a sharp instrument on the head, and on the same side of the head. No sound ever betrayed a struggle, and even people who occupied adjoining rooms knew nothing of the murders. All the bodies were dragged into the back yard and left there in the same position. Such incidents set one thinking of stories like Grant Allen's narrative of mysterious crime called "Habbakkuk J. Jephson's Statement," which most people thought when they read it was an effort of imagination in the region of the improbable.

It may interest our readers to know that they can procure a copy of "The Illness of the Emperor Frederic III.," published in Berlin in English, by forwarding a money order for 1*s*. 10*d*. to Messrs. A. Asher and Co., 5, Unter den Linden, Berlin, W., or to any other well-known firm there. Despite the announcements in the papers, we have found it impossible to purchase copies of this book in England so far.

At the beginning of the winter season, when the fires were first lighted in the Asylum Chapel at Berry Wood, Northampton, it was found that the flues of the heating apparatus would not "draw." This was put down to dampness, and a further trial made, but without success. The smoke obstinately refused to follow its wonted course. A search was made into the internal economy of the apparatus by making an opening near the foot of the chimney, and the following strange collection was brought to light. There were fifty-seven starlings, two jackdaws, one sparrow, a large quantity of twigs and sticks, and five pieces of rope. One of the latter was six feet long, and it is a mystery how the little creatures managed to get this to the top of the chimney before dropping it down. Of course all the birds were dead when found, having evidently got down the flue through the cowl, and, like other animals, found it easier to descend than ascend.

A terrible warning to swindlers comes in a story from Pesth. In a certain town in Hungary there existed a Committee of Hospital Organisation, which for two years had imitated the example given in matters charitable by the mythical Snark, which "collects, though it does not subscribe." These gentlemen had collected funds for a hospital which they had not yet begun to build, when an announcement was received that the Archduke Charles Louis would visit the institution in two days. What was to be done? The committee must have been formed of men of infinite resource, for they at once set to work, hired a home, hired beds, hired nurses, and—last but not least essential—hired patients. When the Archduke arrived they had everything that goes to make a hospital, and his Royal Highness inspected, and approved, and departed well pleased. Then the committee sought to disband the hospital. But the hired patients would not be disbanded. They liked their quarters. The hospital beds were soft, the hospital fare was good, and they were ready—nay, determined—to remain patients for the rest of their days. Finally, they were forcibly ejected, but —and here comes the moral—they brought their case before the eye of the law, and, still as patients of course, had the satisfaction of extracting "damages" for eviction from the ingenious committee. Perhaps the real hospital will get built now.

Total abstinence appears to be gaining ground at the East End of London. It is stated that in a mixed company there it is not the abstainer who has to apologise for his principles, but the man or the woman who drinks wine. A still more striking result of the movement is, that the lions and the lambs of Conservatism and Liberalism lie down together in the most amiable way. Which are the lambs and which the lions does not appear. It is certain, however, that Sir Charles Russell spoke at a Mile End temperance meeting the other day, and quoted Lord Randolph Churchill with approval. Lord Randolph (said Sir Charles) was a man of ability, who had popular pulses stirring within him, who could be counted upon to know which way the cat would jump, and who had drawn attention to the necessity and value of temperance. Sir Charles declared that he would not appear under false colours ; that though he spoke at a temperance meeting, he was not an abstainer. A charitable enthusiast thereupon expressed the fervent hope that he "soon would be."

